# Gallbladder Volvulus Presenting as Acute Appendicitis

**DOI:** 10.1155/2015/629129

**Published:** 2015-06-15

**Authors:** Zachary Bauman, John Ruggero, John Lim

**Affiliations:** Henry Ford Macomb Hospital, Clinton Township, MI 48038, USA

## Abstract

We encountered a case of gallbladder volvulus in an 88-year-old thin female in which the initial presentation was more consistent with that of acute appendicitis. After complete work-up, including physical exam, lab work, and computed tomography, the definite diagnosis of gallbladder volvulus was not made until intraoperative visualization was obtained. Gallbladder volvulus is a rare but serious condition, which requires a high clinical suspicion so prompt surgical intervention can be undertaken.

## 1. Introduction

Gallbladder volvulus (GV), also known as gallbladder torsion, is a rare cause of acute abdominal pain and an uncommon surgical emergency. First described by Wendel as a “floating gallbladder” in 1898 [1], gallbladder volvulus is defined as the rotation of the gallbladder on its mesentery along the axis of the cystic duct and cystic artery [2]. A systemic review published in 2012 concluded that GV is a disease of the elderly and women with a median age of 77 years and a female-to-male ratio of 4 : 1 [3]. Furthermore, there is an associated mortality rate of 6% for patients who develop GV [3].

The common contributing factor for GV is an abnormality in the anatomy of the gallbladder vascular pedicle [4]. Basically, a long or wide mesentery, which includes the cystic artery and duct, can result in torsion of the gallbladder around this axis [4–6]. As people age, visceral fat is often lost. This loss accompanied with liver atrophy can result in lengthening of the gallbladder mesentery and is why GV has a higher incidence in the elderly [2, 4]. Unfortunately, GV can be very difficult to diagnose preoperatively oftentimes presenting as an acute abdomen resulting in emergent surgery [4]. Furthermore, depending on the location of the gallbladder on clinical exam and work-up imaging, a GV can mimic that of acute appendicitis or ischemic bowel [7, 8].

Throughout the years GV has been described on multiple occasions in the literature. We report a case of GV in an elderly female, who was initially suspected to have acute appendicitis given her clinical presentation and laboratory findings. Such a case of GV mimicking acute appendicitis in the elderly has only been reported two other times in the literature [7, 8].

## 2. Case Report

An 88-year-old female was initially admitted to the hospital for hypoxemia secondary to community acquired pneumonia (CAP) and acute chronic obstructive pulmonary disease (COPD) exacerbation. Her past medical history was only significant for COPD and she had never had any previous surgical intervention. She lived at home at the time and had a remote history of smoking. Upon admission, she was treated with appropriate antibiotics and aggressive pulmonary toilet. Her white blood cell count (WBC) upon admission was 18.3 K/*μ*L and by hospital admission day #9, her WBC had come down to 9.9 K/*μ*L and her respiratory status was dramatically improved. The patient was getting ready to be discharged when later that night she suddenly developed right lower quadrant abdominal pain and therefore the surgical service was consulted.

**Figure 1:**
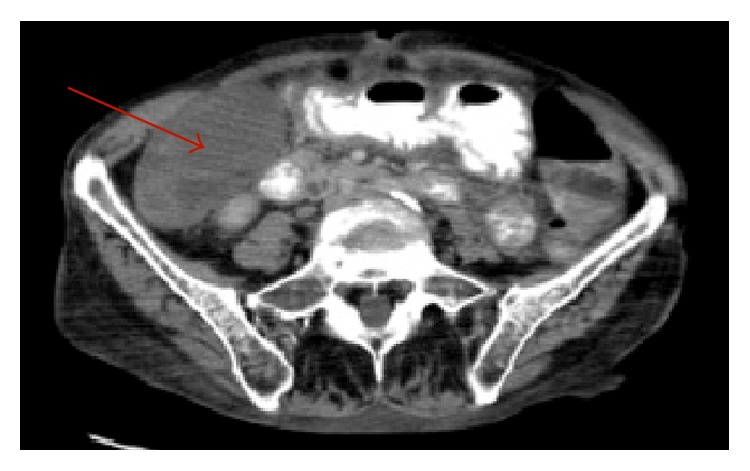
CT scan abdomen and pelvis. Image showing a large “fluid collection” in the right lower quadrant of the abdomen.

**Figure 2:**
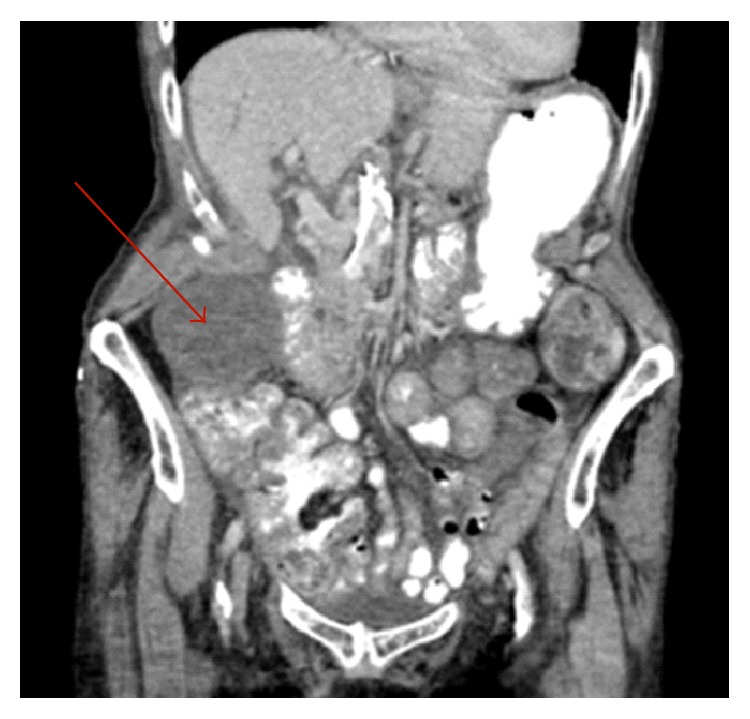
CT scan abdomen and pelvis. Image showing a large “fluid collection” in the right lower quadrant of the abdomen.

The patient was evaluated by surgery on hospital admission day #10. By the time surgery evaluated the patient, she had four bowel movements, one of which was loose in nature, and she stated her abdominal pain was much improved. Physical exam did reveal abdominal pain with light or deep palpation in the right lower quadrant, directly over McBurney's point. An abdominal X-ray was obtained which was unremarkable and new labs were sent at this time. The complete blood count (CBC) did show an acute elevation of the WBC to 16.4 K/*μ*L. Because of this acute elevation in WBC and concern for appendicitis on physical exam, we ordered a computed tomography (CT) scan of the abdomen and pelvis. The CT scan demonstrated an 8.2 cm × 4.8 cm heterogeneous “fluid collection” within the right lower quadrant. An abscess could not be ruled out from the imaging nor could acute cholecystitis. The appendix did appear normal however. Figures 1 and 2 demonstrate the identified fluid collection on CT scan.

**Figure 3:**
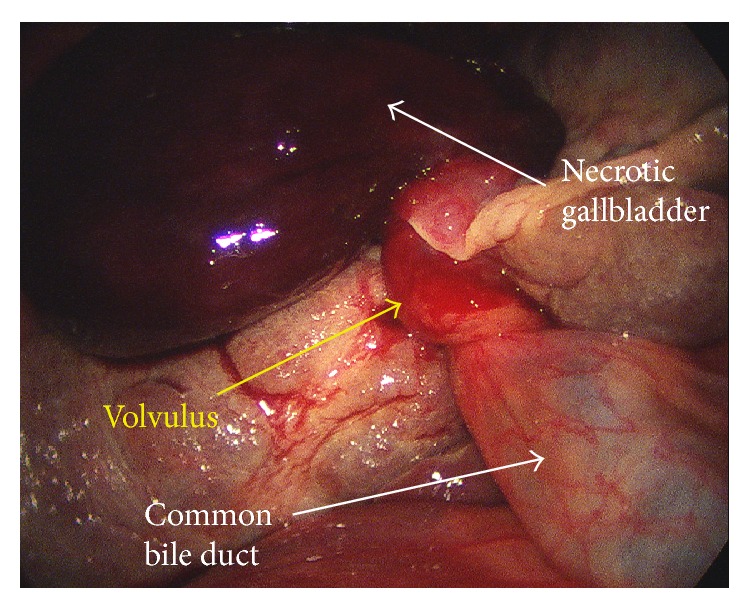
Intraoperative image of gallbladder torsion. Intraoperative picture showing the acute torsion of the cystic duct and cystic artery.

At this point, the patient's antibiotics were adjusted to cover for cholecystitis, appendicitis, and an intra-abdominal abscess as we did not know the exact pathology at this point. She was scheduled for surgery, which was performed laparoscopically. Initially, we placed a 5 mm Visiport trochar just superior to the umbilicus and a laparoscope was inserted. Much to our surprise, the previously described “fluid collection” on CT scan was actually a very distended, necrotic gallbladder that was torted a full 360° in the counterclockwise direction (Figure 3). Luckily the gallbladder had not perforated at this time. Because it was acute gallbladder disease without gross contamination of the abdominal cavity, we were able to perform the cholecystectomy in the traditionally described laparoscopic approach. The volvulus was reduced and the gallbladder was removed through identification of the critical view and ligating the cystic duct and artery.

Postoperatively the patient did very well. She was started on a diet shortly after her surgery and her pain remained well controlled. The pathology report revealed acute gangrenous cholecystitis. The patient was discharged to a rehab center on postoperative day #2 due to some deconditioning she developed from her underlying pneumonia. At the patient's follow-up appointment in the clinic three weeks after surgery, she was found to be doing remarkably well.

## 3. Discussion

Over 750,000 cholecystectomies are performed annually in the United States [9]; however torsion of the gallbladder resulting in volvulus is extremely rare and has a reported incidence of only 1 in 365,000 cases of gallbladder disease [7]. The diagnosis of GV is surprisingly a challenge for both surgeon and radiologist, especially in the preoperative period. After a review of the current literature, only about 9.8% of all GV patients are actually diagnosed preoperatively using all diagnostic modalities available [10, 11]. This was absolutely the case with our patient given the fact that her pain was in the right lower quadrant and the CT scan was inconclusive for gallbladder torsion.

In an attempt to help make the diagnosis of GV preoperatively, Lau developed what has become known as the “Triad of Triads” in 1982, which is used to help identify the clinical features of GV [12, 13]. Triad 1 typically describes patient appearance: elderly, thin body habitus and chronic chest disease or spinal deformity [12, 13]. Triad 2 describes patient symptoms: the typical right upper quadrant pain, sudden and early onset of this pain, and early onset emesis [12, 13]. Finally, Triad 3 illustrates the physical signs of the patient: palpable right upper quadrant mass, lack of a toxic or jaundiced appearance, and a discrepancy between the pulse and temperature [12, 13]. Although this system was designed to identify patients with GV in an easier way, our case only demonstrated 4 out of the 9 previously described characteristics again making the diagnosis of GV in our patient very difficult.

Delay in the diagnosis of gallbladder torsion can result in serious complications that include gallbladder necrosis, gangrene, and subsequent perforation resulting in peritonitis and prolonged hospital stay [7, 8]. Ultrasonography frequently reveals evidence suggestive of “cholecystitis” which includes gallbladder wall thickening and pericholecystic fluid [3, 14]. CT scan of the abdomen usually demonstrates the presence of the gallbladder outside the fossa and inferior to the liver with pericholecystic fluid and a massively distended gallbladder with thickened walls [14–16]. Furthermore, gallbladder torsion upon completion of hydroxy iminodiacetic acid (HIDA) scan shows a “bullseye” image secondary to accumulation of the radioisotope in the gallbladder [3, 17]. Despite all these highly technical imaging modalities, GV is still not usually discovered until surgical intervention [14], which was indeed the case in our patient as the CT scan was not fully conclusive of gallbladder torsion.

In conclusion, GV should remain on the differential diagnosis especially if there are multiple criteria for the “Triad of Triads” met or the patient has radiographic evidence of GV. Laparoscopic cholecystectomy remains the current treatment option of choice [4, 7, 18] rendering early diagnosis of GV important to avoid a more invasive approach. Previous cases report the recovery after a laparoscopic approach to torsion of the gallbladder is only 2-3 days [19, 20], which was definitely the case with our patient. As stated previously, GV can be very challenging to diagnosis preoperatively, which was the case with our patient as we were under the impression that she had acute appendicitis given her clinical picture. A high level of suspicion should be maintained by all members of the treatment team when working up patients with a gallbladder volvulus.
